# Reliability Assessment of Wireless Sensor Networks by Strain-Based Region Analysis for Redundancy Estimation in Measurements on the Example of an Aircraft Wing Box

**DOI:** 10.3390/s24134107

**Published:** 2024-06-24

**Authors:** Sören Meyer zu Westerhausen, Gurubaran Raveendran, Thorben-Hendrik Lauth, Ole Meyer, Daniel Rosemann, Max Leo Wawer, Timo Stauß, Johanna Wurst, Roland Lachmayer

**Affiliations:** Institute for Product Development, Leibniz University Hannover, An der Universität 1, 30823 Garbsen, Germanylachmayer@ipeg.uni-hannover.de (R.L.)

**Keywords:** wireless sensor network, system reliability, measurement redundancy, region growing algorithm, finite elements, strain analysis

## Abstract

Wireless sensor networks (WSNs) are attracting increasing research interest due to their ability to monitor large areas independently. Their reliability is a crucial issue, as it is influenced by hardware, data, and energy-related factors such as loading conditions, signal attenuation, and battery lifetime. Proper selection of sensor node positions is essential to maximise system reliability during the development of products equipped with WSNs. For this purpose, this paper presents an approach to estimate WSN system reliability during the development phase based on the analysis of measurements, using strain measurements in finite element (FE) models as an example. The approach involves dividing the part under consideration into regions with similar strains using a region growing algorithm (RGA). The WSN configuration is then analysed for reliability based on data paths and measurement redundancy resulting from the sensor positions in the identified measuring regions. This methodology was tested on an exemplary WSN configuration at an aircraft wing box under bending load and found to effectively estimate the hardware perspective on system reliability. Therefore, the methodology and algorithm show potential for optimising sensor node positions to achieve better reliability results.

## 1. Introduction

**Figure 1:**
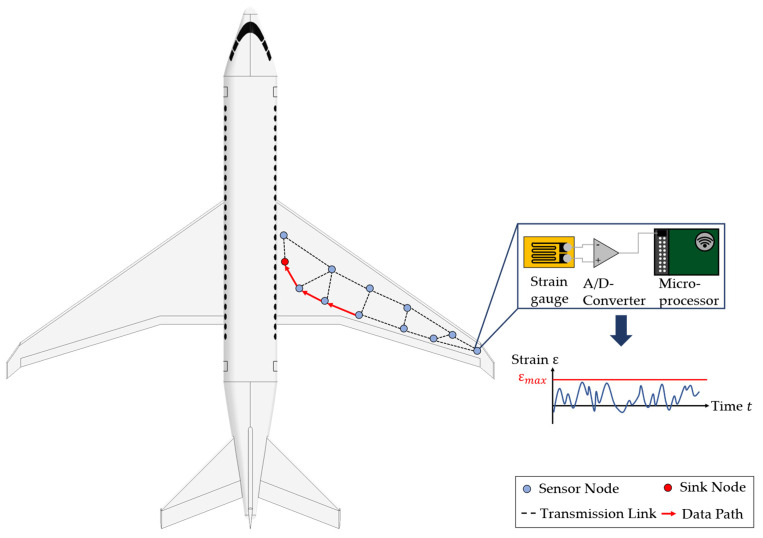
Example of a wireless sensor network and a data path for data transmission from one sensor node to another along defined transmission links (adapted from [11]).

Using sensor networks (SNs) for monitoring large areas of load-carrying structures and components is a well-known technology which is used in various applications, like, e.g., structural health monitoring (SHM) of bridges [1] and airplane structures [2] or the condition monitoring of wind turbines [3]. In these cases, the sensors of the WSN enable the remote monitoring of damages and, therefore, to make decisions regarding the remaining useful life or for scheduling condition-based maintenance [4]. In Figure 1, an exemplary WSN is shown for the purpose of monitoring an aircraft wing. The sensor nodes can be used in this case to monitor the structural behaviour, e.g., the strain field of the wing. The data are then transmitted along defined transmission links to the sink node. A further analysis of the collected data allows to find damage positions and sizes [5]. Moreover, the data of the strain field could be used to gain insights into the loading history of the aircraft wing [6]. This allows to draw conclusions for optimisation potentials in the development of the next generation of the wing regarding the design for the operational loads, which is called product generation development [7]. Especially when the sensors or the WSN are directly integrated into the component, like, e.g., into the layup of a composite, the load information becomes an integral part of it and is “inherited” from one product generation to the next. Therefore, such components are called gentelligent in the paradigm of technical inheritance, which was investigated in detail in the Collaborative Research Centre 653 [8,9]. Considering the aircraft wing in Figure 1 as a gentelligent component, the WSN fulfils its function by measuring the strains over time. For the purpose of getting information on the bending of the wing, the sensor nodes are aligned on two parallel lines, as in the study of Valoriani et al. [10].

In a general form, each sensor node consists of one or more sensors and, if needed, an analogue-digital converter (A/D converter) and a microprocessor, which has a module for a wireless connection for data transmission to other sensor nodes [11]. For the depicted case, strain gauges are used as sensors for measuring. Their analogous signals are converted to digital signals with the A/D converter and are then processed at a microprocessor and transmitted via its wireless module. The links and the data paths result from specified transmission protocols, which are explained in Section 2.1 in more detail. In the case of the WSN shown in Figure 1, it becomes clear that the reliable fulfilment of the data collection and transmission function depends on a lot of different components in the network as well as the way data are transmitted. Therefore, different challenges regarding the reliability of a WSN have to be considered. For example, data transmission itself, along with data delivery [12] or sensor node battery lifetime [13], are often referred to in the literature. However, from an engineering design perspective, the use of WSN brings further challenges when high-quality data should be acquired over a long period of time. For example, in SHM systems in aerospace applications, the whole system must survive different harsh environmental and operational conditions for around 20 years. So, the system service life should be comparable to the life of the monitored component or should be designed as repairable [14]. But besides this hardware point of view on the lifetime, the WSN reliability is also determined by its capability to ensure the required data quality. Different authors, therefore, addressed the topic of reliability in data acquisition processes with WSN from different points of view on hardware lifetime, energy consumption and data transmission, as reviewed in [15]. Most publications in the field of reliability in WSNs focus on the data point of view by addressing the problems of data loss, data delay, and data accuracy, and energy consumption is often considered [15].

The system reliability from a hardware lifetime point of view is, however, rarely addressed in the literature. So, a methodology is presented for the automatic assessment of a WSN’s system reliability from a hardware lifetime point of view based on region analysis from FEM simulation data. This methodology can be considered as a follow-up of the works presented in [11,14]. Therefore, the paper is structured as follows: In Section 2, an overview of related work on algorithms for the automatic reliability assessment of sensor networks and on algorithms for region analysis to find measuring regions with redundant data is given. Afterwards, the methodology developed for this paper is described in Section 3. To show the applicability of the developed methodology, its applicability is demonstrated in a case study in Section 4. Lastly, Section 5 concludes this paper and gives an outlook on further research on this topic.

## 2. Related Work on Wireless Sensor Networks, Reliability Modelling, and Region Analysis

The use of WSNs in various applications makes it necessary to estimate the reliability for each case using the right methods. Therefore, general knowledge about the network configuration and the methods for the calculation of the system’s reliability are necessary. This section provides a short overview of WSNs, the data transmission in them and the principle of established transmission protocols (Section 2.1). Furthermore, an overview of methods for the calculation of the system reliability is given in Section 2.2, which is the basis of related works in Section 2.3, presenting methodologies to calculate the system reliability of WSNs. Section 2.4 gives an overview of related works to the estimation of measurement regions for the algorithm used in this paper.

### 2.1. Data Transmission in Wireless Sensor Networks

**Figure 2:**
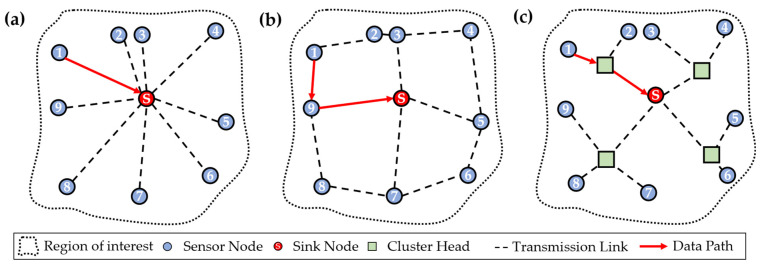
Comparison of resulting network configurations due to the resulting transmission links from the (**a**) DIRECT, (**b**) FLOODING, and (**c**) LEACH protocol (adapted from [11,14,16]).

When data acquired by the sensor nodes in a WSN have to be transmitted, the path for data transmission is determined by the used transmission protocol. In the following, the three basic forms of transmission protocols, DIRECT, FLOODING, and LEACH, are considered. At this point, it should be noted that these are only generic terms for those data transfer protocols since they have many other subtypes. The schemes for data transmission are depicted in Figure 2 for each of these three transmission protocols. In each of the schemes, a resulting data path from sensor node ‘1’ to the sink node is shown to highlight the differences in the transmission protocols.

Especially when transmissions are not of great distance, the DIRECT protocol could be used (see Figure 2a). All the sensor nodes have only one transmission link, along which data are transmitted directly from the measuring sensor node to the sink node, where data are gathered and further sent to the end user. The scheme of this network architecture shows that the unweighted sensor nodes form a graph in the form of a so-called star topology around the sink node. The use of this protocol could result in less network administration and organisation but might cause higher energy consumption for data transmission along greater distances. This issue is especially important when the WSN is battery-powered and should still be working independently for a long time [13]. When the distances for data transmission are greater, and there is no possible connection between some sensor nodes and the sink node, or the energy consumption for data transmission is too high due to the long distances, the FLOODING transmission protocol could be used. As shown in Figure 2b, data are sent from one sensor node to the other hop-by-hop until it arrives at the sink node. Therefore, data paths with multiple hops might result in this protocol. However, each of the sensor nodes is still of the same weight regarding the hierarchy in the network [17]. In case the LEACH protocol, shown in Figure 2c is used, there are additional sensor nodes as so-called cluster heads (CHs) added to the network. To each CH, a group of sensor nodes is assigned. Therefore, data from assigned sensor nodes are gathered and then transmitted as a whole to the sink node. From this architecture, a hierarchal tree structure is derived, where the sink node has the highest weight, the CHs form the next level in the hierarchy, and the sensor nodes are at their lowest level. Such structure might be useful when a more complex network is considered with a high amount of sensor nodes, where the data transmission requires more organisation than in the other two cases [16].

In the following, only the two transmission protocols, DIRECT and FLOODING, will be considered further since the focus of this work is on use cases where the WSNs are less complicated and complex, so the use of the LEACH protocol is not sensible.

### 2.2. Calculation of the Reliability of Non-Repairable Wireless Sensor Networks

In the literature, different models for analysing the system reliability of a WSN can be found. These models can be distinguished into models for repairable and models for non-repairable systems. Repairable systems are considered, for example, by Ibrahim et al. with a Markov chain approach [18] and by Li and Huang with a Petri net approach [19]. In these models, WSNs are analysed regarding their availability with different repair rates for the battery, for example. However, when considering WSNs without a battery as a power supply, the repairability of components in the WSN becomes secondary since the other components have a relatively long lifetime when considered under normal operational conditions and with no corrosive environmental influences. Therefore, only methods for the reliability assessment of non-repairable WSNs are considered in the following.

**Figure 3:**
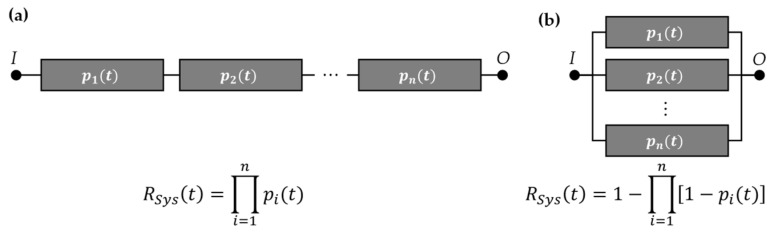
Reliability block diagram (RBD) in the two basic forms: (**a**) series and (**b**) parallel order with the corresponding equations to calculate the system reliability.

In the field of system reliability of non-repairable systems, the fault tree analysis (FTA) and the reliability block diagram (RBD) are commonly used methods. Considering an RBD, the different failure mechanisms of the elements of the system are represented by blocks. These elements connect the system input *I* and output *O*. To ensure the proper work of the system, a connection between *I* and *O* has to be always present. If this is not the case, a system is considered as failed [20]. For the reliability-oriented representation of a system using an RBD, there are mainly three configurations distinguished: (1) the series order, (2) the parallel order, and (3) the combination of both. The equations to describe the system reliability of the first two basic forms are displayed with their graphical representation in Figure 3.

In the first form of a series order, all of the independent elements are needed to work to fulfil the system’s function. The probabilities of functioning elements at a time *t*, which is equal to their reliability ($p_{i}\left(t\right)=R_{i}(t)$), are combined by multiplications. So, the system reliability is calculated as the product of each block’s reliability. In the case of a parallel-ordered RBD, the connection between *I* and *O* is present as long as one block is still working. Therefore, each element’s probability of failure ($q_{i}\left(t\right)=F_{i}(t)$) is multiplied to calculate the system’s probability of failure. With the connection $F_{i}\left(t\right)=1-R_{i}\left(t\right),$ the calculation of the system reliability results are obtained, as shown in Figure 3 [13].

### 2.3. Reliability Modelling of Non-Repairable Wireless Sensor Networks

In the field of reliability engineering, fault tree analysis (FTA) and the reliability block diagram (RBD) are commonly used methods to analyse the reliability of a non-repairable technical system [20]. In the work of Wang et al. [21], a dynamic FTA is used to investigate different fault-tolerant mechanisms in the reliability analysis of a WSN. In their work, they considered different backup strategies of sensor nodes in a whole WSN. The study considered the cases of cold and hot backup of single-type sensors, the reliability analysis of sensors for hot backup in homogenous and heterogenous as well as hybrid forms, and the reliability analysis of sensors for cold backup in homogenous, heterogenous, and hybrid forms. However, the result that a cold backup yields a higher reliability than a hot backup and that a heterogenous form yields higher reliability than a homogenous scheme is not surprising. Furthermore, it only focuses on the combination of different sensors as a network instead of considering a SN as a more complex system consisting of different sensor nodes, which are composed of sensors, microcontrollers, Wi-Fi modules and so on.

A focus on a whole sensor node as a part of the network consisting of more than sensors is set in the 2013 study by Distefano et al. [22]. In this work, the reliability of a WSN is analysed with a focus on the mean time to failure (MTTF) for three network topologies. A dynamic reliability block diagram (DBRD) is used for this purpose to take the operation modes “Active” and “Sleep” of sensor nodes into account. The distinction between these modes allows to take different energy consumptions into account when a sensor node is, for example, not transmitting data. Furthermore, this approach allows to take different energy consumptions resulting from the number of connections and, therefore, data transmissions due to the network topology into account. A similar approach was chosen by Nuhu et al., where the energy consumption of a sensor node in an SHM application was measured with and without sleep mode [23]. With these data, the reliability of a sensor node is calculated in dependence on the time in months and decades in a simulation.

The topic of energy consumption as a reliability criterion is also taken into account by Gurupriya and Sumathi in their 2022 work on multipath routing algorithms for WSN [24]. Besides the energy consumption, they considered the average data delay in dependence on the number of nodes in the network for their reliability estimation as well. Therefore, simulations of data transmission were carried out with a battery discharge model for reliability analysis.

To calculate the reliability of a whole WSN, the reliability model needs to describe the connections of network components, as well as a detailed analysis of the lifetime of single sensor nodes and their battery lifetime, data transmission and so on. Therefore, the approach presented by [25] could be used. In their work, a WSN is modelled as a graph *G* = (*V*, *E*), consisting of nodes *V* and edges *E*. When the graph is assumed to be built by *n* sensor nodes, forming a star topology, the network reliability can be calculated, as shown in Equation (1).

(1) $$R_{G}\left(t\right)={\left[R_{V}\left(t\right)\right]}^{n}\times {\left[R_{E}\left(t\right)\right]}^{n-1}$$

**Figure 4:**
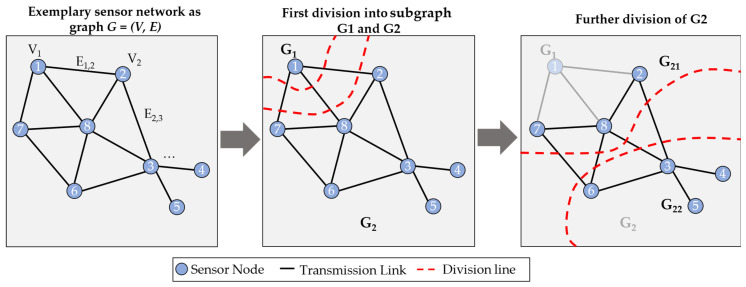
Example of the functionality of network division into star topologies by Lin et al. (adapted from [26]).

Therefore, the system reliability is defined as a series order of the nodes and edges in a reliability block diagram (RBD). However, the system reliability is more difficult to calculate when the WSN is forming a mesh, as when the FLOODING protocol is used and the sensor nodes have more than one connection to one another. In that case, the number of edges can be higher than the number of nodes. To calculate the system reliability in that case, Lin et al. assume that every graph *G* could be divided into different subgraphs *G_i_* until each subgraph forms a star topology. This division is exemplary shown in Figure 4. In the first step, the graph *G* = (*V*, *E*), consisting of *V* = 8 nodes and *E* = 11 edges, is divided into two subgraphs, *G*_1_ and *G*_2_. As one can see, subgraph *G*_1_ could not be divided any further, and, therefore, one can calculate its reliability according to Equation (2), assuming each node has the same reliability $R_{V}\left(t\right)$ and each edge $R_{E}\left(t\right),$ respectively.

(2) $$R_{G1}\left(t\right)=R_{V}\left(t\right)\times \left[1-{\left(R_{E}\left(t\right)\right)}^{3}\right]$$

In Equation (2), the term for the edges as path reliability forms a parallel order of the three edges divided in this step into the subgraphs RBD. This is due to the fact that the data from node ‘1’ is transmitted redundantly along these three links. If one or even two of them fail, the data could still be transmitted.

A further division of subgraph *G*_2_ leads to the subgraphs *G*_21_ and *G*_22_ in Figure 4, where each forms a star topology. Therefore, the system reliability of each subgraph could be calculated the same way as for subgraph *G*_1_. However, the main issue of this approach is that redundancies of sensor nodes due to measurements are not taken into account. Assuming that node ‘1’ and node ‘7’ would measure the same values of the monitored physical phenomenon, there would also be a redundancy of these nodes, which would not be detected in this approach.

**Figure 5:**
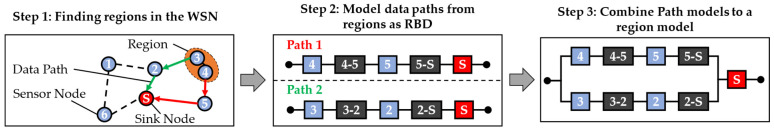
Process of building region models as RBD from data paths of sensor nodes in a WSN (adapted from [11,14]).

This issue is overcome by Dâmaso et al. in their 2014 study [13]. In this work, a WSN is not just divided by the location of sensor nodes and the network topology but by their measurements. It is assumed that sensor nodes form regions when they measure the same physical phenomenon, which is not further specified in more detail. After a region is identified, the data paths of each sensor node inside the region are analysed. In the methodology defined by Dâmaso et al., each data path is modelled as a series order RBD with each sensor node and transmission link along which the data are transmitted. This is exemplary shown in Figure 5, where the sensor nodes ‘3’ and ‘4’ form a region together.

After modelling the data paths as RBD, they are combined into a region model, as depicted in Figure 5. In the region model, the data paths form a parallel structure in the resulting RBD until they are combined at a specific point along their way, as shown in Figure 5 for the data paths from nodes ‘3’ and ‘4’, which form a parallel order until they are combined after their links to the sink node, which is then connected as a series element. The parallel order results from the redundancy of the nodes since they are measuring redundantly. In case one of the nodes fails or the data transmitted do not arrive at the sink, the region is still monitored by the other node. Only the sink node is a series element in since, in case of a failure of it, data will no longer be sent to the user.

However, even this approach has some disadvantages since it only takes the battery lifetime into account as a reliability criterion, and the connection of region models to a WSN system model is not specified. To take the methodology a step further and overcome this issue, the work of Dâmaso et al. was applied and modified by Meyer zu Westerhausen et al. [26] for the application of WSNs with the only purpose of strain measurements. Therefore, the physical phenomenon as a criterion for the region analysis is the strain amplitude at the different measurement areas. If sensor nodes are positioned close to each other and measure the same strain with consideration of the measurement tolerance, they are considered redundant sensor nodes. In case the sensor nodes are far away, for example, at different sides of a component, or the measured strains are too different, no redundancy is detected. This approach was presented on the example of virtual strain measurements from a finite element (FE) model of a simulation based on the finite element method (FEM). Furthermore, the approach presents a way to calculate the system reliability of the whole WSN instead of only the reliability of the regions as subsystems. This calculation of the system reliability is performed under the assumption that each region is relevant for the measurement task. Therefore, a failure of one region would lead to a failure of the whole system. From this, it follows that the system reliability is calculated as a series order of the regions in an RBD [26].

The methodology presented by Meyer zu Westerhausen et al., however, suffers from two drawbacks. First, the calculation of the reliability of sensor nodes and links is based on the consideration of only sensor nodes and links, but it neglects different failure modes on the component level of the sensor nodes. So, the RBD of the regions has to be more detailed by modelling the sensor nodes as an RBD on the component level. Second, there is a potential risk of inaccuracies regarding the region analysis. Assuming sensor nodes are redundant only by a user-specified radius around them might lead to false results in the reliability analysis when the radius is assumed to be too big or too small. Furthermore, regions with similar strain values do not necessarily be circular. This might lead to inaccuracies when a sensor node measures the same strain due to the size of a region with similar values but is out of range from another node. Therefore, other approaches for the region analysis have to be taken into account.

### 2.4. Algorithms for Region Analysis

The issue of identifying similar regions is not only a reliability analysis issue but also in the area of image analysis. Since rectangular two-dimensional finite elements, as used in the approach of [26], can be seen as analogous to pixels in images, the application of region analysis techniques from the image analysis field seems promising. Therefore, different algorithms for region analysis from these fields are presented below.

**Figure 6:**
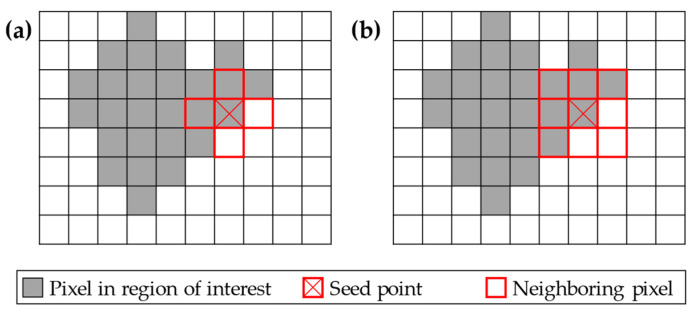
Example for the segmentation of an image into regions of interest with the region growing algorithm by (**a**) the 4-neighbour and (**b**) the 8-neighbour approach (adapted from [27]).

In various applications and publications, especially in medical applications for analysing computer tomographic (CT) images, the region growing algorithm (RGA) became of interest. In this context, the pixels in the image are compared regarding their greyscale level, for example, to find the boundaries of tumour tissue to the healthy environment [27]. Additionally, the algorithm could also be used for the segmentation of colour images in multimedia applications, as demonstrated by Ikonomakis et al. [28]. However, the general procedure of the RGA consists of the following four steps. In the first step, one pixel of the image is chosen as the seed point, and its neighbouring pixels are taken into the set $N_{S}$ of this initial state of an image segment. If one of the neighbouring pixels $p\in N_{S}$ fulfils the homogeneity criterion H, for example, a specific deviation from the seed point’s greyscale value is not exceeded, it is marked, and its neighbouring pixels are also taken into the set $N_{S}$. If one of the initial neighbouring pixels does not fulfil H, it is marked as processed, and it is continued with the next pixel. Therefore, no neighbouring pixels are considered for the segmentation process for region identification if H is not fulfilled. This procedure is repeated until there are no further neighbouring pixels fulfilling the homogeneity criterion [27]. For a better explanation, the process is depicted in Figure 6, where a region of interest (ROI) is shown in grey, and the seed point is marked with a red X. The neighbouring pixels are marked with a red border.

As one can see, there are two possible approaches for neighbour selection in this two-dimensional (2D) case. In Figure 6a, the neighbouring pixels are selected in each iteration step with a 4-neighbour or direct neighbour approach. Therefore, the pixels have to share a common edge. In contrast, for the 8-neighbour approach in Figure 6b, it is only necessary to share a common node to classify two pixels as neighbours [27,29,30].

The RGA in the described form is applied, modified and enhanced in various publications. For example, in the publication of Mešanović et al., the RGA is applied for the purpose of computer-aided diagnosis for lung diseases is tailored to the application on CT images with the use of a histogram analysis to identify the threshold values between a “risk area” and the surrounding tissue. Therefore, it is able to identify different regions within the same slice of a CT image [31]. A human lung is also analysed by Wang and Li in their study from 2022. In their work, the RGA was applied for tumour identification from sliced CT images. For this purpose, the automatic choice of seed points for the RGA was adapted to incorporate the use of prior knowledge on lung tumours. This enhanced the segmentation accuracy of the tumour region identification, which underlines the importance of choosing appropriate positions for the seed points in RGA applications [32].

Besides greyscale images like the ones derived from CT, the RGA also has the potential to identify objects from coloured images. This is investigated in the work of Jain and Susan, where eleven cases of different objects are analysed with different algorithms, where an adapted RGA with the use of the 8-neighbour approach is applied. The results of the study, therefore, showed that the RGA has the potential to be applied in cases with more varying factors to consider for the homogeneity criterion [30].

The RGA is also applied in fields besides medical and object detection applications, like the analysis of images taken of areas for geographic purposes using satellites or planes. For example, Pan and Fang applied the RGA in the analysis of overlapping parts of images to cut the images along seamlines, which were identified with improved seeding for the RGA. Therefore, it is possible to connect different pictures after cutting into a mosaic, forming a picture without overlapping regions [33]. In another application presented by Kang et al., the RGA is applied on a three-dimensional (3D) point cloud from laser scanning of a tunnel. Therefore, the 3D point cloud is cut into 2D slices, where the points are used to form masks, where regions are identified. These identified regions from the segmentation are used in the RGA again to identify adjacent, overlapping slices. As a result of this, a point cloud can be derived, where features (e.g., tubes along the tunnel) are identified and displayed in the same colour as connected regions [34].

All the above-mentioned publications have in common to be follow-ups or adaptions of the RGA in its basic form, which are only adapted to the examples used. However, it becomes clear with a focus on the region analysis’ purpose for reliability analysis, as described in Section 2.3, that there are two main differences between the RGA in the presented forms: The state of the art considers the RGA only for sliced parts of an image or a point cloud, but not whole 3D models and the RGA is not applied to FEM models and simulation results. Therefore, the region analysis for the use of identification of measurement regions, as described in [26], is not emphasised enough in the literature under consideration of the RGA for this purpose.

## 3. Methodology for Reliability Analysis Based on Region Analysis of FEM Simulations

**Figure 7:**
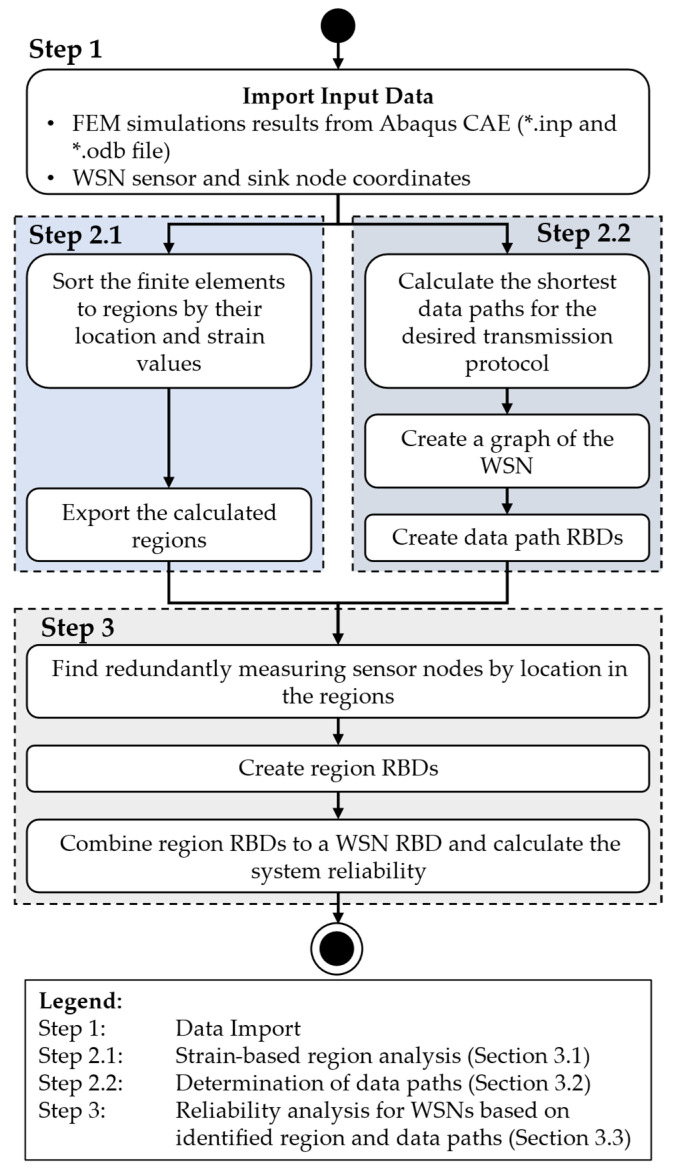
Process for the application of the proposed methodology.

As presented in Section 2.3, there are different approaches with different focuses on the topic of reliability analysis of WSNs. From the presented approaches, it becomes clear that the methodology presented in [26] is suitable for the calculation of the system reliability of WSNs with the purpose of strain monitoring. Therefore, it is further developed and improved, considering an application for system reliability analysis during product development of component-integrated WSNs. To overcome the identified issues in the methodology, the improved form is presented in the following. In this methodology, the RGA from image analysis (see Section 2.4) is adapted for analysing FEM simulation results to split the component under consideration into regions by the strain data. Afterwards, redundantly measuring sensor nodes are identified. The calculation of the WSN system reliability is then performed like in [26] but with a more detailed reliability model of the sensor nodes. This model considers each element of the sensor node separately instead of the sensor node as a whole. A general overview of the process the user has to apply for using this methodology is shown in Figure 7. In this process, the user has to choose and import simulation data from an FE model as well as the information on sensor and sink node locations for the planned WSN.

Regarding the FEM simulation data, the Abaqus CAE Standard/Explicit formats *.inp and *.odb, as well as an Abaqus CAE Standard/Explicit licence, are required. With these data, the adapted RGA, published as Python code on GitHub [35], is applied to sort the elements of the FE model by their strain data and location to regions and export this information. These steps are described in Section 3.1 in more detail. Parallel to this part of the process, the sensor and sink node coordinates are used with the information of the planned transmission protocol to calculate the shortest paths for data transmission from each sensor node to the sink node, resulting in a graph model of the WSN. To calculate the shortest paths, Dijkstra’s algorithm [36] is applied. From this graph, the RBDs of the data paths are derived, like in the approach of [11,14]. This part of the methodology is explained in Section 3.2 in more detail.

By the combination of each sensor node’s coordinates and the exported regions, redundantly measuring sensor nodes are identified. This allows to derive the region RBDs, in which the data path RBDs are combined, if a measurement redundancy is found. The region RBDs are then combined to a WSN-RBD to calculate the system reliability of the WSN. This part of the process is described in Section 3.3 in more detail.

### 3.1. Strain-Based Region Analysis for FEM Results to Estimate Measurement Redundancies

At the beginning of the before described methodology, FEM simulation data are imported for the step of the strain-based region analysis. Therefore, the element definition by the nodes and node coordinates is imported, as well as the data of the strains in the different directions of each element. Moreover, the tolerance of the strain gauges planned to be used for the sensor nodes has to be defined, as well as the information if the 4- or the 8-neighbour approach should be used for the region analysis. The use of these two approaches leads to a limitation of the methodology since it can only be applied for two-dimensional quadrilateral elements, which have to be linear because quadratic elements do not only have nodes on the corners of the elements, which disables the analogy of the finite element to a pixel in an image. For a better understanding of the implementation of the FEM simulations adapted RGA, referred to as RGA4FEM in the following, it is shown in Algorithm 1 in pseudocode.

First, after the import of the element and node definitions as well as the strain results, the algorithm checks which elements are potential neighbours by their definition using the node indices of each element. To do so, the contact criterion has to be fulfilled, so in the case of the 4-neighbour approach, two shared nodes at the same edge are required, or in the case of the 8-neighbour approach, one shared node is required. This check is performed for every possible combination of two elements, *i* and *j*, and results in a contact matrix, where geometric neighbouring elements are marked as TRUE, or as ‘1’ respectively.

After the geometric contacts of elements are defined in the contact matrix, the region analysis starts based on the strain of each element as the homogeneity criterion. Therefore, an initial seed point is set. From this seed point, the neighbouring elements from the contact matrix are analysed regarding the fulfilment of the homogeneity criterion. This means that only elements with a strain difference less than the measuring tolerance of the strain gauge and a strain value that is in tolerance to the region’s average are added to the region. If one of these criteria is not fulfilled, the element is marked as processed for this region and will be used as a seed point for a new region. This step is repeated until all elements are assigned to a region.
**Algorithm 1.** Strain-based RGA for FEM simulations (RGA4FEM)
**Input:***.inp and *.odb file, neighbouring approach, measurement tolerance
**Output:**List and *.inp file with elements clustered into sections by the regions1Read the *.inp file and store node and element definitions in lists2Export strains of each element from the *.odb file3Combine strain results with list of element definitions in one list4**for** element **in** element list **do:**5  **if** element *i* **contacts** element *j*:6    Set contact of element *i* and *j* on TRUE in the matrix7  **else**:8    Set contact of element *i* and *j* on FALSE in the matrix9**end**10Store contact information in a matrix11Set initial seed point for region analysis12**While** not all elements assigned to regions **is TRUE**:13  **if** strain difference of elements in contact is less than the measuring tolerance
  **and** strain value of the current element is less than the region average:14    Add element to region15  **else**:16    Set element as next seed point of another region17**end**18Save regions with assigned elements to a list19Write a *.inp-file with regions as sections for post-processing in Abaqus

**Figure 8:**
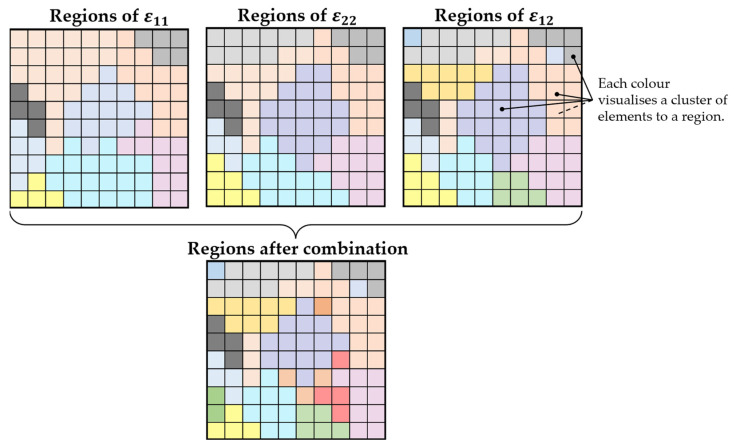
Exemplary sketch of shell elements combined into regions and the derivation of a region segmentation after the combination of the regions for different strain directions.

This region analysis could be repeated for each strain direction exported from Abaqus. For two-dimensional shell elements, this means the strains $\varepsilon_{11}$ and $\varepsilon_{22}$ in the main directions, as well as $\varepsilon_{12}$ in the shear direction. Strains in the direction of the element thickness ($\varepsilon_{13},\;\varepsilon_{23}$ and $\varepsilon_{33}$) cannot be considered because of the missing thickness of shell elements. As depicted in Figure 8, these regions in different strain directions can be used to derive one list of regions and sections due to the combination of the element assignment. In this figure, elements with the same colour form a region together. For the different strain directions, these assignments might differ. This leads to a finer segmentation of the FE model when the different region assignments are compared and combined.

**Figure 9:**
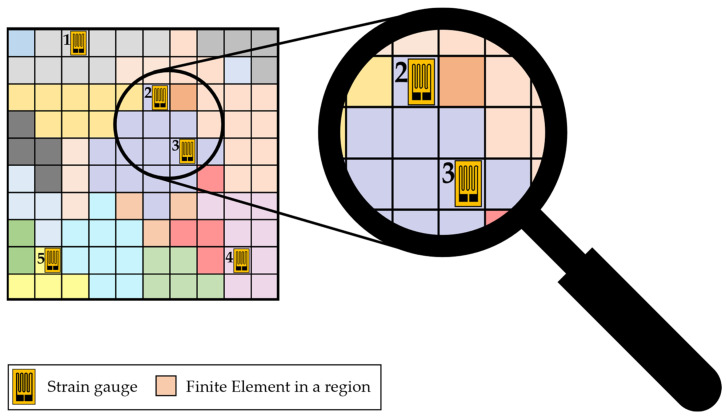
Example of elements from the FE model with five strain gauges positioned on them with a measurement redundancy of strain gauges ‘2’ and ‘3’.

After the region analysis is finished, the resulting FE model with the segmentation of elements into sections by their regions is used to estimate measurement redundancies for given sensor positions. For this purpose, the needed coordinates for the sensor nodes or, respectively, the sensors are imported first. Afterwards, it is analysed in which regions the sensors are placed and this assignment of the sensors to the corresponding regions is saved in a list, which is used for further redundancy estimation. If two sensors are detected to be placed in the same region, they are assumed to measure redundantly since the strains in the regions differ only in a range which is below the measurement tolerance of the sensors. An example is depicted in Figure 9.

In this example, five strain gauges are positioned as sensors by their imported coordinates. It is observable that the strain gauges ‘1’, ‘4’, and ‘5’ are the only sensors in their regions, which could be determined by the different colours of the elements, whereas the strain gauges ‘2’ and ‘3’ are positioned in the same region. Therefore, a measurement redundancy of strain gauges ‘2’ and ‘3’ is present. Using this region-based approach allows therefore, to detect measurement redundancies easily due to the assignment of elements to region. For example, a sixth sensor at the element directly above strain gauge ‘5’ would not lead to redundancy, even though the elements are in contact, since their strains differ more than the sensor’s measurement tolerance and are therefore assigned to different regions.

### 3.2. Determination of Data Paths in the WSN

**Figure 10:**
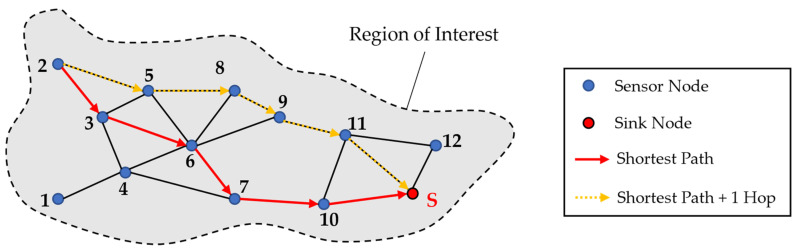
WSN in the region of interest with links between the sensor nodes resulting from the FLOODING protocol with the shortest path and the shortest path added with one additional hop from sensor node ‘2’ to the sink node.

Parallel to the region analysis with the aforementioned RGA4FEM algorithm, a network configuration regarding the data paths is created using the imported coordinates of sensors and sensor nodes. Furthermore, it is important to determine which data transmission protocol should be used. In the following, only the DIRECT and the FLOODING protocol are considered. Furthermore, the maximal transmission range of the sensor nodes is required for this task. For the determination of all the data paths resulting from the DIRECT protocol, leading to links between each sensor node and the defined sink node, a directed graph *G* is created. For this task, the algorithm loops through the list of sensor nodes, creates a directed edge from the node to the sink node, and saves this path to a path list. If it is not possible to create a link between a sensor node and the sink node due to the limited transmission range, the algorithm ends with an error. In case the FLOODING protocol is chosen, the algorithm creates a directed graph *G*, where each sensor node is connected to all the sensor nodes in range. The estimation of suitable data paths is carried out using Dijkstra’s shortest path algorithm [36]. In Figure 10, an exemplary WSN is shown, where the possible links resulting from the FLOODING protocol are shown between the twelve sensor nodes and the additional sink node.

In the exemplary WSN, data are transmitted along the shortest path from sensor node ‘2’ along the nodes ‘3’, ‘6’, ‘7’, and ‘10’ until they arrive at the sink with five hops. However, in case a node or a link fails along the data path, it could be useful to create additional data paths to send data redundantly. For this purpose, a path which is not the shortest path can be chosen with, for example, one additional hop. Therefore, in this methodology, additional paths with more hops can be chosen to be added, like it is depicted in Figure 10 with the dotted yellow arrows.

### 3.3. Reliability Analysis for WSNs Based on Identified Regions and Data Paths

For the reliability analysis after the steps before, it is assumed that a sensor node consists of only one sensor, in this case, a strain gauge, an analogue–digital converter (A/D converter), cables connecting them, a microprocessor with a built-in transceiver for data transmission and cables connecting the A/D converter to the microprocessor. Since each of these elements is required to allow the function of the sensor node, these elements form a series order in an RBD for reliability calculation. Therefore, the reliability of a sensor node is calculated as

(3) $$R_{SensorNode}\left(t\right)=R_{StrainGauge}\left(t\right)\cdot R_{Cable}\left(t\right)\cdot R_{A/D-Converter}\left(t\right)\cdot R_{Cable}\left(t\right)\cdot R_{Microprocessor}\left(t\right).$$

For this calculation, it is required to obtain the Weibull parameters of each component of the sensor node defined by the user for the same loading condition. Furthermore, it is necessary to obtain the Wöhler curve slope factor defined by the user for each component to allow the calculation of the reliability under consideration of different load amplitudes. Regarding these inputs, the following three assumptions become clear for the presented methodology:

1. The lifetime of sensor nodes is considered independent of their energy consumption and the battery lifetime due to the assumption of an unlimited energy supply.
2. Fatigue is considered here as the only failure for a sensor node under cyclic loading conditions due to the high dependence of the reliability of solder joints for cables and for the signal drift and failure of strain gauges due to cyclic loads [37].
3. The Weibull shape parameter *b* does not change for each component since the failure and material are considered equal for each component of the same category.

**Figure 11:**
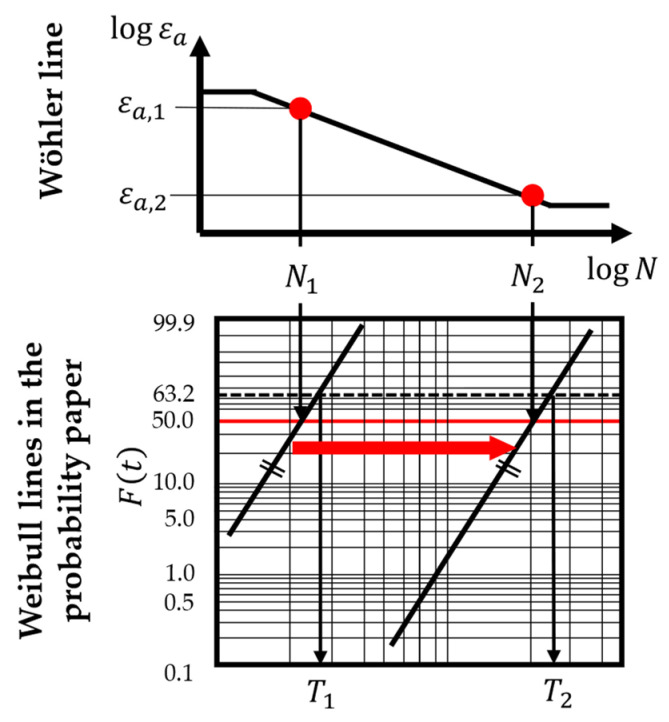
Parallel displacement of a Weibull line in a probability paper along the 50% failure probability line to estimate the scale parameter *T* for different points on the Wöhler line.

As the shape parameter *b* of the Weibull distribution describing the reliability of each component does not change, the scale parameter *T* has to be calculated for each loading condition at the different points for each sensor node. For this purpose, the Wöhler line slope factor *k* has to be defined as well for each component of a sensor node. Since the focus of this paper is on strain measurements, it is assumed that the *k*-factor is determined for different strain amplitudes. Moreover, the Wöhler line describes a failure probability of 50% at each point of it. Therefore, the Weibull distribution describing the failure characteristics of a component can be determined for each point on the Wöhler line for a known reference point on it, its slope factor *k*, and the Weibull shape parameter *b* (see Figure 11).

Since point ‘1’ on the Wöhler line with the strain amplitude $\varepsilon_{a,1}$ for $N_{1}$ cycles has a failure probability of 50 %, it is found at the Weibull line in the Weibull probability paper at the time in load cycles $N_{1}$ and $F\left(t\right)=50\%$. Therefore, the Weibull scale parameter for this loading condition $T_{1}$ can be estimated. To obtain the scale parameter for another loading condition with a strain amplitude $\varepsilon_{a,2}$ for point ‘2’ on the Wöhler line, the Weibull line in the probability paper has to be moved parallel to its origin (marked with the red arrow in Figure 11) until it intersects the point with $F\left(t\right)=50\%$ at a time of $N_{2}$. This parallel displacement is only possible under the assumption that the failure and the material of the component under consideration stay the same. For the moved Weibull line, it is possible to estimate the new corresponding scale parameter $T_{2}$ for this loading condition. However, this procedure is implemented in the presented methodology using Equation (4).

(4) $$T_{2}=\frac{N_{1}\cdot {\left(\frac{\varepsilon_{a,1}}{\varepsilon_{a,2}}\right)}^{k}}{\sqrt[{b}]{\ln\left(\frac{1}{0.5}\right)}}$$

In this equation, $\varepsilon_{a,1}$ and $N_{1}$ mark the reference point on the Wöhler line, $\varepsilon_{a,2}$ is the strain amplitude at the considered sensor node position, k is its slope factor, and b is the Weibull shape parameter. The factor of 0.5 in this equation results from the 50 % failure probability, which connects the Wöhler line and the corresponding Weibull distribution.

To calculate not only the reliability of a single sensor node but of a whole data path, all sensor nodes and their links form a series-ordered subsystem of the whole WSN. The series order results from the fact that each sensor node or link is needed in this path for the data transmission, and, therefore, a failure of one of these elements will lead to a failure of the whole data path.

**Figure 12:**
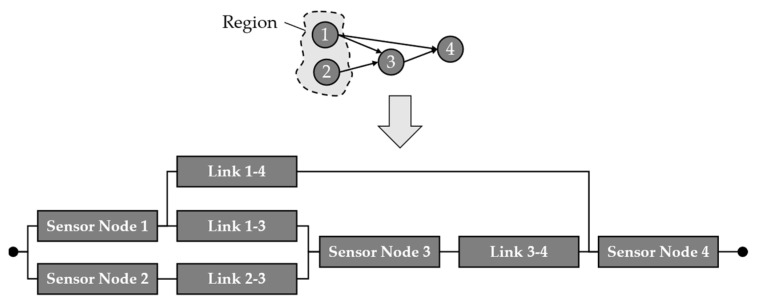
Example of a region in a wireless sensor network and the resulting reliability block diagram for the sum of disjunct paths used in the reliability analysis.

However, as described in the methodology (see Section 3), the data paths are combined into a region model based on the redundancies detected during the region analysis (see Section 3.1). This combination might lead to complicated structures in the RBD, which could not be calculated based on the equations for simple series or parallel structures. A structure, as shown in Figure 12, requires a calculation of the reliability based on the sum of disjunct paths (SDP). For this calculation, Equation (5) is used, where $P_{i,dis.\;j}$ is the reliability of an element i in the disjunct path j.

(5) $$R_{path}=\sum_{i=1}^{m}\left(\prod \left\{P_{i,dis.\;j}\right\}\right)$$

Figure 12 shows an example of a region and the data transmission from it as a source to the target at sensor node ‘4’. From these data paths, the shown RBD is derived for the calculation of the region’s system reliability regarding data acquisition and transmission. Therefore, the disjunct paths are calculated as paths of success. For this purpose, the minimal paths ${MP}_{i}$ are determined first as $MP_{1}=\left\{N_{1},L_{14},N_{4}\right\},\;MP_{2}=\left\{N_{1},L_{13},N_{3},L_{34},N_{4}\right\},$ and $MP_{3}=\left\{N_{2},L_{23},N_{3},L_{34},N_{4}\right\}$, where $N_{i}$ are the sensor nodes and $L_{ij}$ are the links between them.

The reliability of the region $R_{region}$ built by sensor nodes ‘1’ and ‘2’ is then calculated, as shown in Equation (6), applying the SDP method. In this equation, $\bar{R_{MP,i}}$ denotes the negotiation of the reliability of the path.

(6) $$\begin{array}{c} R_{region}=R_{MP,1}+\bar{R_{MP,1}}\cdot R_{MP,2}+\bar{R_{MP,1}}\cdot \bar{R_{MP,2}}\cdot R_{MP,3}\\ =R_{N,1}\cdot R_{L,14}\cdot R_{N,4}\\ +\bar{R_{N,1}\cdot R_{L,14}\cdot R_{N,4}}\cdot R_{N,1}\cdot R_{L,13}\cdot R_{N,3}\cdot R_{L,34}\cdot R_{N,4}\\ +\bar{R_{N,1}\cdot R_{L,14}\cdot R_{N,4}\cdot R_{N,1}\cdot R_{L,13}\cdot R_{N,3}\cdot R_{L,34}\cdot R_{N,4}}\cdot R_{N,2}\cdot R_{L,23}\cdot R_{N,3}\cdot R_{L,34}\cdot R_{N,4} \end{array}$$

**Figure 13:**
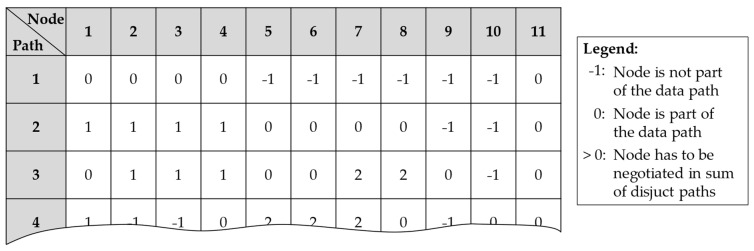
Part of the matrix for calculating the reliability of a region with the SDP method.

For the automatic calculation of these disjunct paths for a whole sensor network, the above-described process is implemented into an algorithm, which is based on the algorithm of Heidtmann to calculate the SDP [38]. As input, the data paths of a region from Section 3.1 and Section 3.2 are required, as well as a corresponding matrix that describes which sensor nodes are included in each path of success. After the algorithm is finished, a matrix is derived as exemplary, as depicted in Figure 13, where each row represents a data path, and each column is a sensor node in the whole WSN.

Every matrix entry with a ‘−1’ shows that this sensor node is not part of the data path, whereas a ‘0’ means that these nodes are included in a path. Furthermore, every entry >0 shows terms which have to be negotiated. Equation (7) exemplary describes the resulting region reliability for the data paths ‘1’, ‘2’, and ‘3’.

(7) $$R_{region}\;=\;R_{1}R_{2}R_{3}R_{4}R_{11}+R_{5}R_{6}R_{7}R_{8}\cdot \bar{R_{1}R_{2}R_{3}R_{4}}+R_{1}R_{6}R_{6}R_{9}R_{11}\cdot \bar{R_{2}R_{3}R_{4}}\cdot \bar{R_{7}R_{8}}+\cdots$$

**Figure 14:**
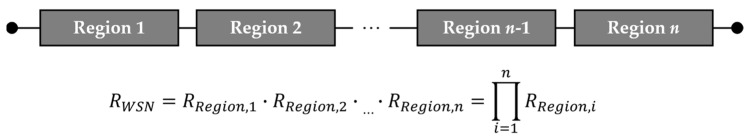
Series-ordered RBD with blocks of each region in the WSN and the calculation of the WSN system reliability as a product of all region reliabilities.

This process of reliability calculation is repeated until the reliability of each region in the whole WSN is described using the SDP method. If this is the case, the reliability of the whole WSN is calculated by multiplication of all region reliabilities since they form a series order in an RBD, as depicted in Figure 14. This calculation is based on the assumption that each region is required to fulfil the measurement task of the network. Therefore, a failure of one region would lead to a failure of the whole WSN and results in a series-ordered RBD.

The results from the calculation of the WSN system reliability from all the region reliabilities could then be further analysed, yielding insights into possible weaknesses regarding the network reliability and the fulfilment of defined requirements. If, for example, one region strongly influences the reliability of the WSN in a negative way, the development engineer could then change the network architecture by adding a redundantly measuring sensor node, which results in higher reliability.

## 4. Case Study of the Proposed Methodology

**Figure 15:**
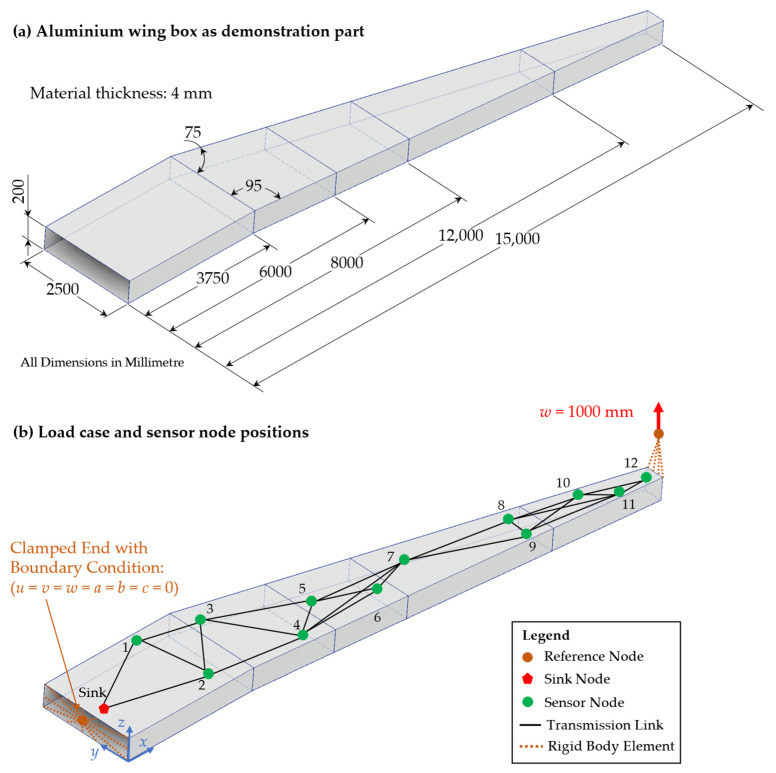
Simplified aluminium wing box as demonstration part for the case study with (**a**) its dimensions and (**b**) the load case used for the simulation, as well as the positions of the WSN.

The methodology proposed and described in Section 3, along with the implementation in algorithms, is tested in the following case study to show its applicability for the region-based reliability analysis of WSNs as whole systems. For this case study, the part shown in Figure 15a with a length of 15 m is used as a demonstrator. This part represents a simplified aluminium wing box of a passenger aircraft, which is part of the wing for stiffening the aerodynamic wing profile. Therefore, the area-wide strain measurement has great potential to show possible structural optimisations for weight savings. For such area-wide measurements and data transmissions, a WSN is used with the goal to measure and transmit the load data reliably during a defined time in the product use phase.

Since an aircraft wing is mostly loaded under bending, the load case, as depicted in Figure 15b, is used for simulation, where a displacement of 1000 mm is applied on a reference node at the tip of the wing. At the other end, the boundary condition of a clamped end with no degrees of freedom in all directions for displacement and rotation is defined at a second reference node. This load case is based on the results of a simulated airplane wing for design optimisation in the work of Wunderlich et al. [39]. The reference nodes are connected to the part using rigid body elements. This allows the application of the boundary conditions defined at the corresponding reference nodes uniformly to the nodes of the discretised part. Both the reference nodes and the rigid body elements are shown in orange in Figure 15b. For the simulation of the above-described load case, the demonstration part is transformed into a midsurface model in Autodesk Inventor 2023 and imported into Abaqus CAE for simulation using the Standard/Explicit 2021 solver. The geometry is discretised using two-dimensional linear shell elements from type S4R with a length of ≈100 mm and a width varying between 200 mm at the clamped end and 35 mm at the tip under load. The boundary conditions (clamped end and displacement at the free end) are defined at the corresponding reference nodes.

Furthermore, the positions of the sensor nodes (green points) and the sink node (red polygon) are marked in Figure 15b, as well as the transmission links connecting them with the use of a FLOODING protocol for data transmission with a transmission range of 3 m. The exact positions with *x*-, *y*-, and *z*-coordinates are listed in Table 1. These positions are chosen based on the above-described idea of using the strain sensor data for the reconstruction of loads and deformations of the wing with sensor nodes positioned on two parallel lines [40], as in the current layout, with a distribution in analogy to [41].

**Figure 16:**
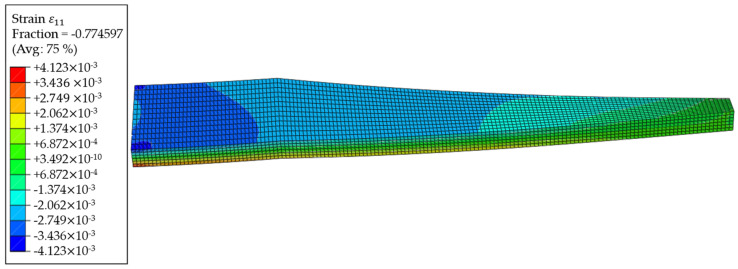
Plot of the strains from the FEM simulation in the length direction of the part $\varepsilon_{11}$.

In Table 1, the positions of the sensor nodes are shown, as well as the strain $\varepsilon_{11}$ amplitude, that results in the length direction of the part at the corresponding positions. Those strains, as well as the element IDs in the model, are exported from the Abaqus output database file (*.odb-file). A plot of these strains $\varepsilon_{11}$ is shown in Figure 16.

**Figure 17:**
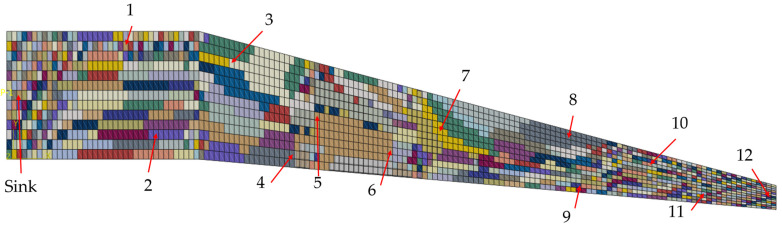
Results from the region analysis using the RGA4FEM with the 8-neighbour approach with marked positions of the twelve sensor nodes and the sink node.

The strains of all elements are exported from the *.odb-file for the region analysis. Furthermore, the elements, as well as their definitions with the nodes, are exported from the Abaqus Input file (*.inp-file) since this information is required to find neighbouring elements, as described in Section 3.1. The application of the algorithm is programmed in Python (see Code at GitHub [35]), and a measurement tolerance of 1% is chosen for the strain gauges, which is a value derived from measurements using a strain gauge in combination with an HX711 A/D converter and amplifier and an Arduino Nano 33 IoT as a microprocessor with an integrated transceiver. Furthermore, a deviation of 5% from the region’s average values is allowed to keep the computational effort for the algorithm in a considerable amount. To show the applicability of the algorithm, the 8-neighbour approach is chosen, resulting in 2197 regions. The regions at the upper surface of the wing box where the sensor nodes are positioned are shown in the plot of Figure 17. The choice of the 8-neighbour approach was made due to the fact that the 4-neighbouring approach tended in before conducted studies to oversegmentation, leading to higher computational effort and smaller regions. Furthermore, the 8-neighbour approach is suitable for models with unstructured meshes since the 4-neighbour approach leads to errors when a combination of rectangular and triangular elements is present in a model.

In Figure 17, the elements forming regions together are shown in the same colour. As one can observe, the regions at the clamped end (left side of Figure 17) are smaller than those in the middle of the part. This is due to the higher variance of the strains at this section of the part since the maximal strain absolute values are observed at the clamped end, and the corners have a higher stiffness (see Figure 16). Beginning at the 11th element row in the length direction, the regions tend to grow larger, showing smaller strain gradients than at the clamped end. However, along the length of the part, smaller regions of one to three elements appear, even in the middle of the length. This happens at the parts of the wing box, where a stiffening panel is mounted inside, leading to changes in the steady strain gradients. Furthermore, a very high segmentation at the free end, where the load in the form of the displacement is applied, could be observed. This high segmentation has two reasons. First, the plot of strains in Figure 16 shows a diagonal progression at this part, where there is only the chance for elements with a shared node at the corner to form a region together. Second, the RGA4FEM checks the strain difference in two neighbouring elements in percent. Therefore, the tolerable deviation between two elements with very low strain absolute values, as it is observed at the free end, is also very low.

Regarding the use of the results from the RGA4FEM for reliability analysis, one can derive a single measurement redundancy from Figure 17. For this purpose, it is necessary to take a look at regions and check if sensor nodes are positioned in a region together. For example, the sensor nodes ‘5’ and ‘6’ measure nearly the same strain since they are positioned inside the same region, even though they have an Euclidian distance of 1580.275 mm. A detailed look at Figure 17 makes clear that these are the only sensor nodes in the same region, so there is no other measurement redundancy inside this WSN.

Following the presented methodology, the next step is to model the data paths of all regions as RBD. Since the FLOODING protocol is used in this example, the data are sent hop-by-hop from the sensor nodes in each region to the sink node. Therefore, data paths result in varying links and nodes along them. Especially for the sensor nodes that are far away from the sink node, the data paths become long. The RBDs for all data paths as shortest paths in the exemplary WSN and the resulting region models are shown in Table 2.

The calculation of each region’s data path system reliability requires knowledge about the shape parameters *b* of the Weibull distribution, describing the failure probability of the components of the sensor nodes as well as the links. In this study, it is assumed that each link has a constant reliability of 100 %, which means that no data loss or link failure is considered, for example, in the 2017 study of Deif and Gadallah [42]. Furthermore, the Wöhler line slope factor *k* is required, which describes the fatigue failure characteristics of the sensor nodes. The values of these parameters are shown in Table 3. For this case study, the *k*-factor results form the HBM handbook for strain gauges describing their signal drift and failure characteristics. It is derived from strain gauge failures at the strain amplitudes at the corresponding failure times in cycles $\varepsilon_{a,1}\left(N_{1}=7\cdot 10^{4}\;cycles\right)=3\;\frac{mm}{m}$ and $\varepsilon_{a,2}\left(N_{1}=2\cdot 10^{6}\;cycles\right)=2\;\frac{mm}{m}$ [43]. The Weibull parameters are assumed to be derived from tests at $\varepsilon_{a,1}$, so the use of Equation (4) allows us to calculate the shape parameters for each other strain amplitude on the Wöhler line.

**Figure 18:**
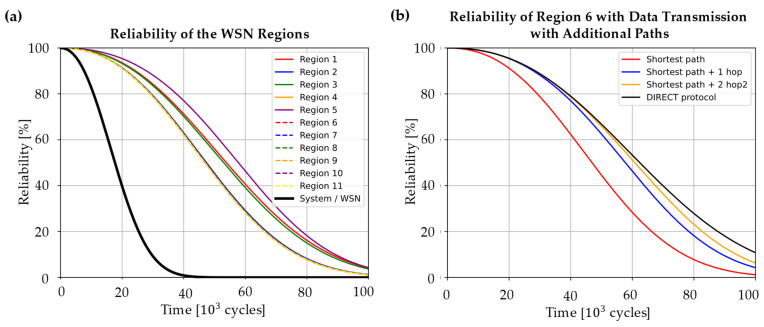
Reliability of (**a**) each region’s data path with one hop and (**b**) region ‘6’ with more hops than the shortest path.

The reliability of the region’s data path results is depicted in Figure 18 for the WSN under consideration. The reliability of each sensor node is calculated under consideration of Equations (3) and (4) and the WSN system reliability, as depicted earlier in Figure 14. For the calculation, a program script in Python was written and used.

In Figure 18a, one can observe clearly that the data path from Region ‘5’ has the highest reliability. This is due to the detected measurement redundancy of the sensor nodes ‘5’ and ‘6’. These two sensor nodes also have very different data paths to the sink node so the data paths from the region form a parallel order in the RBD with the sink node as a series element. Therefore, the reliability is higher than, for example, of region ‘1’. This region has just two elements in series order (sensor node ‘1’ and the sink node), but due to the redundancy of region ‘5’, its reliability is higher. Moreover, the sensor nodes of region ‘5’ do not experience as high loads as sensor node ‘1’ in region ‘1’, which also leads to higher reliability. The combined effect of low loads leading to a longer lifetime of single nodes and a longer path length leading to a decreasing reliability due to more series elements in an RBD could be well observed in the example of region ’11’. Even though the strain amplitude at this node is with $\varepsilon_{a}=3.1\cdot 10^{-4}$ comparatively low, the long data path from the wing tip to the wing root leads to the lowest reliability of the whole network. Since all the regions form a series order in the RBD of the whole WSN, the system reliability is much lower than even the reliability of region ‘11’.

If the system reliability is too low for the planned application, changes in the physical architecture and/or the data transmission itself are needed. If, for example, the WSN system reliability should be 80 % at 20·10^3^ cycles, it could be observed that this is not reached with the current configuration. Changes in the data transmission might be carried out more easily since there is no completely new WSN to be planned. Therefore, considering redundancies in data transmission as well, more hops could be chosen from specific sensor nodes. In Figure 18b, this is exemplarily considered for region ‘6’, where data are transmitted from sensor node ‘7’ to the sink node. As one can see, with the number of data paths considered besides the original shortest path, the reliability of the region’s data path increases. However, the gain of reliability is limited due to the fact that sensor node ‘7’ and the sink node still form critical elements in the RBD, which limits the reliability of their value due to the series order. Therefore, the more hops are considered, the more the reliability converges to the value the region would have with one hop when the DIRECT data transmission protocol is used since, in this case, the reliability is also limited by the series order of the source and the target node in a series order. However, the longer transmission range would, in contrast, lead to a higher energy consumption, as described by Dâmaso et al. [44].

**Figure 19:**
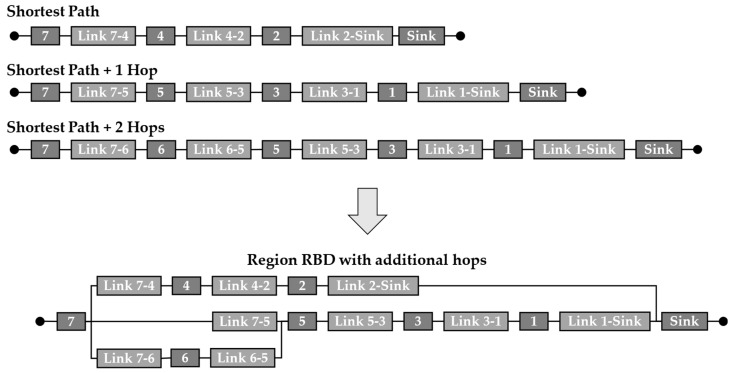
RBDs for the shortest data path in region ‘6’ and the data paths with additional hops.

The increasing number of data paths with partly overlapping path segments makes the use of the sum of disjunct paths (SDP) necessary since the reliability could not be calculated with simple series and parallel orders in the region RBD. Figure 19 shows an exemplary RBD for the reliability of region ‘6’ with consideration of additional data paths with a greater length and more hops than the shortest path. Due to the more complicated structure of the RBD, the system reliability is calculated using the SDP.

As it becomes clear from Figure 19, the visualisation and effort for the reliability calculation for the regions increases when more hops are considered. However, the calculation still works fast due to the automated calculation with the SDP. Additionally, the RBD is only used as a visualisation method for the calculation, making it not necessary for the application of the presented methodology.

## 5. Discussion

The applicability of the methodology described in Section 3 was presented in the example of a WSN mounted on the top surface of a wing box as part of an aircraft wing. For this purpose, the presented RGA4FEM was applied to the results for the strains of the wing box under bending load. The results of this region analysis showed similarities to the progression of the strains in the colour plot of the deformed geometry, which underlines the algorithm’s capability to properly sort elements to regions when they fulfil the homogeneity criterion regarding the strain values. However, it could be observed that the wing box has a geometry where parts of it are not arranged directly along the axis of the coordinate system, leading to possible inaccuracies when considering the strain in its length direction. This issue could be overcome when the part under consideration is oriented in a way that the measurement direction of the strain gauges fits the direction of the coordinate system axis. Moreover, the algorithm is limited to 2D shell elements in its present form, which limits the applicability to thin-walled components. However, it is working well for such models, even though they are geometrically complicated, like the wing box with its stiffening elements.

Regarding the reliability analysis, the presented methodology presents a suitable follow-up on works in the state of the art with a focus on non-repairable WSNs that allows for estimating possible weaknesses regarding the data transmission in the network. Especially the combination of the approaches of Dâmaso et al. [14] and Meyer zu Westerhausen et al. [11] seems promising from the case study’s results since the implementation of the sum of disjunct paths (SDP) used by Dâmaso et al. allows for enhancing the approach of Meyer zu Westerhausen et al., as presented. This allows us to consider data transmission with more than one data path from a sensor node to the sink node, leading to better analysis results regarding the consideration of transmission redundancies. But it becomes clear from the example of more data paths under consideration that the RBD as a modelling technique is limited for visualisation of the data paths from the reliability perspective. This is because it might get confusing and overloaded for long data paths and a lot of linked elements. However, the RBD is only a tool for visualisation, so it does not make the methodology less applicable.

With a focus on the correctness of the results of the reliability analysis, one can say that the results in the case study are as expected since sensor nodes with a low loading condition regarding the strain amplitude have a longer lifetime than those under higher loads. But it also becomes clear that these sensor nodes have, in the presented case study, a longer distance to the sink node, which leads to decreased reliability due to more elements in series order in the RBD. This result fits the expected results since the system reliability decreases faster when a system with more elements in series order is considered in contrast to systems with only a few series-ordered elements. In the case study, this becomes especially clear when region ‘1’ and region ‘11’ are compared, where region ‘1’ has a data path with only two nodes included, whereas the data path of region ‘11’ includes seven nodes (see Table 2 in Section 4). Even though the sensor nodes of region ‘1’ are under higher load regarding the strain amplitude, which leads to a faster-decreasing reliability, the regions’ reliability is higher than the one of region ‘11’ (see Figure 18a in Section 4).

Since the purpose of the presented methodology is to help engineers design WSNs that fit the reliability requirements of the part which should be monitored, it has to clearly show optimisation potentials. For example, in the case study, region ‘11’ could be identified as a part of the WSN, which leads to a lower system reliability. This might be addressed by the definition of additional data paths for transmissions, as it becomes clear when additional data paths with more hops are considered (see Figure 18b). Therefore, the methodology has the potential to aid in the design process of such WSNs. However, for a design that considers not only the hardware perspective on reliability, the methodology has to be enhanced regarding the data and energy perspective on the WSN reliability. To take these into account, the presented methodology has the potential to be, therefore, enhanced since the modelling of the data paths might allow considering, for example, changes in the signal quality and, therefore, the data accuracy. For this purpose, the signal attenuation during data transmission could be added and analysed for each hop along every data path of regions. The required energy consumption for the data transmission along the data paths could also be added to this analysis. This would then lead to a better understanding of weaknesses in the network due to a more holistic view of the WSN system reliability.

## 6. Conclusions and Future Work

The use of wireless sensor networks (WSNs) allows us to fulfil measurement tasks with distributed sensors in wide-area applications. The application is not limited to large areas in civil engineering and geoscience since WSNs are also applied for monitoring in mechanical and aerospace engineering. Especially for the development of structural large-scale components, the measurements give valuable insights into the load histories of parts and, therefore, point out possibilities for design optimisations for future product generations. When structural components in the field of aerospace applications are considered, this might lead, for example, to reduced weights and, therefore, fewer fuel consumptions since design optimisations might remove unnecessary material. In modern, smart product applications, the sensors and sensor networks become integral parts of the components. So, when a WSN, therefore, becomes such an integral part of the structure itself, it has to work reliably for the whole product life since it cannot be changed or repaired after the integration inside the FRP layup. Therefore, the system reliability has to be analysed before the component is manufactured so that it fits the requirements of the component. For this purpose, the literature presents different strategies for the reliability analysis. Most of the publications are, however, focused only on energy consumption as a limiting factor of a WSN’s lifetime. The data paths are often analysed regarding the energy consumption during data transmission, or methodologies for reliability analysis of repairable systems are considered, which are not applicable for structure-integrated WSNs due to the missing access into the part itself. When methodologies are not mainly focussed on this perspective on the reliability of a WSN, the analysis of the system reliability often does not allow us to draw conclusions on which parts of the WSN might lead to issues regarding the reliability and need to be optimised to fit the requirements.

In this paper, a methodology is presented which allows us to analyse the system reliability of a WSN based on the data paths that are necessary to fulfil the measurement and data transmission function. This methodology includes measurement redundancies in the reliability analysis with a region growing algorithm (RGA), which is adapted to the analysis of finite element (FE) models and the strains resulting from the FE analysis. This adapted RGA is therefore referred to as RGA4FEM, from which sensor nodes in the same regions with similar strains are found as redundantly measuring. Each region’s data paths are then modelled as reliability block diagrams (RBDs) for non-repairable systems, using the sum of disjunct paths (SDP) for the reliability calculation. The SDP allows the calculation of the reliability of data paths, which could not be reduced into simple series and parallel orders, which is especially of interest when more than the shortest path is considered for data transmission. To show the applicability of the methodology, a case study is presented, where the reliability of an exemplary WSN with data transmission using the FLOODING protocol is analysed on the example of a wing box as a stiffening element of an airplane wing. In this case study, two sensor nodes were found to work redundantly, leading to the highest reliability of all regions measured in the WSN. Furthermore, the influence of additional data paths with an increasing number of hops during data transmission besides the shortest path is demonstrated.

The discussion of the results showed that there is still potential for future work on this methodology. For example, the methodology analyses the data paths and has, therefore, great potential to integrate the reliability of data transmission regarding package loss, too late delivered packages, and decreasing accuracy due to data transmission range and, therefore, decreasing signal power. The modelled data paths also enable us to include the energy perspective when the energy consumption for data transmission is considered. Therefore, models for the battery lifetime and models for the discharge have to be added, as well as the effect of energy consumption systems. This will be an improvement of the presented methodology and lead to a holistic approach to the reliability analysis of WSNs from the hardware, data, and energy perspective. Regarding the calculation of the hardware reliability, more failure possibilities besides failure due to fatigue should be included. Adding these enhancements will be part of future works on the presented methodology. Furthermore, the RGA4FEM will be enhanced so that models with volume elements can also be analysed, and the chance of an oversegmentation is reduced to the inclusion of suitable criteria for minimal detectable strains. It is also considered to combine the presented methodology and algorithm with an optimisation algorithm for the problem of optimal sensor placement from a reliability point of view. One possibility might be the application of a genetic algorithm for multifactorial optimisation, where sensor nodes are placed in the regions, and the different resulting configurations form individuals for the optimisation. Each of the individuals in the generations would then be evaluated in a fitness function regarding the maximisation of the reliability from each of the three perspectives. Moreover, the minimisation of the number of sensor nodes might also be a goal, but it has to be performed with the consideration of keeping redundant working sensor nodes when needed from a hardware point of view.

## Figures and Tables

**Table 1:** Positions of the sensor nodes and their strain amplitude from the FEM simulation.

Node	Element ID	x [mm]	y [mm]	z [mm]	$\mathrm{Strain}\;\varepsilon_{11}$
Sink	5311	246.71	1250.00	600.00	−2.82 × 10^−3^
1	5579	2319.08	2211.54	600.00	−2.70 × 10^−3^
2	5666	2911.18	480.77	600.00	−2.81 × 10^−3^
3	4626	4385.81	1870.93	600.00	−2.43 × 10^−3^
4	4461	5559.72	92.45	600.00	−2.37 × 10^−3^
5	3223	6047.55	841.68	600.00	−2.32 × 10^−3^
6	3025	7476.12	166.05	600.00	−2.22 × 10^−3^
7	2683	8449.91	541.80	600.00	−2.12 × 10^−3^
8	2362	10,949.87	432.26	600.00	−2.02 × 10^−3^
9	2326	11,149.87	−513.03	600.00	−1.38 × 10^−3^
10	147	12,532.10	−99.55	600.00	−1.61 × 10^−3^
11	296	13,596.58	−666.79	600.00	−7.26 × 10^−4^
12	462	14,854.55	−723.49	600.00	−3.10 × 10^−4^

**Table 2:** Shortest data paths from the sensor nodes to the sink node for data transmission with the FLOODING protocol and 3 m transmission range with the region RBDs.

Region	Node	Shortest Path to Sink	Region RBD
1	1	1 – Sink	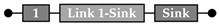
2	2	2 – Sink	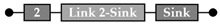
3	3	3 – 1 – Sink	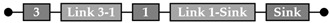
4	4	4 – 2 – Sink	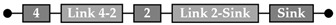
5	5	5 – 3 – 1 – Sink	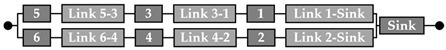
	6	6 – 4 – 2 – Sink	
6	7	7 – 4 – 2 – Sink	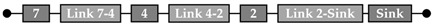
7	8	8 – 7 – 4 – 2 – Sink	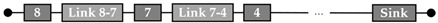
8	9	9 – 7 – 4 – 2 – Sink	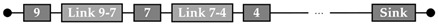
9	10	10 – 8 – 7 – 4 – 2 – Sink	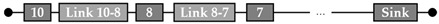
10	11	11 – 8 – 7 – 4 – 2 – Sink	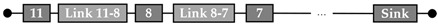
11	12	12 – 10 – 8 – 7 – 4 – 2 – Sink	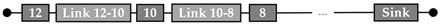

**Table 3:** Parameters needed for the calculation of the reliability of relevant parameters.

Part	Weibull Shape Parameter *b*	Wöhler Line Slope Factor *k*
Strain gauge	2.0	8
A/D converter	2.3	10
Microprocessor	3.1	12
Cable	2.7	7

## Data Availability

All the data used and generated for this publication are available from the corresponding author. Developed software is not available due to privacy restrictions.
